# Mass drug administration with azithromycin for trachoma elimination and the population structure of *Streptococcus pneumoniae* in the nasopharynx

**DOI:** 10.1016/j.cmi.2020.07.039

**Published:** 2021-06

**Authors:** Rebecca A. Gladstone, Ebrima Bojang, John Hart, Emma M. Harding-Esch, David Mabey, Ansumana Sillah, Robin L. Bailey, Sarah E. Burr, Anna Roca, Stephen D. Bentley, Martin J. Holland

**Affiliations:** 1) Parasites and Microbes, Wellcome Sanger Institute, Hinxton, UK; 2) Medical Research Council Unit The Gambia at LSHTM, Fajara, Banjul, Gambia; 3) London School of Hygiene & Tropical Medicine, Keppel Street, London, UK; 4) National Eye Health Programme, Ministry of Health and Social Welfare, Kanifing, Gambia

**Keywords:** Azithromycin, Mass drug administration (MDA), Pneumococcus, *Streptococcus pneumoniae*, Trachoma

## Abstract

**Objective:**

Mass drug administration (MDA) with azithromycin for trachoma elimination reduces nasopharyngeal carriage of *Streptococcus pneumoniae* in the short term. We evaluated *S. pneumoniae* carried in the nasopharynx before and after a round of azithromycin MDA to determine whether MDA was associated with changes in pneumococcal population structure and resistance.

**Methods:**

We analysed 514 pneumococcal whole genomes randomly selected from nasopharyngeal samples collected in two Gambian villages that received three annual rounds of MDA for trachoma elimination. The 514 samples represented 293 participants, of which 75% were children aged 0–9 years, isolated during three cross-sectional surveys (CSSs) conducted before the third round of MDA (CSS-1) and at 1 (CSS-2) and 6 (CSS-3) months after MDA. Bayesian Analysis of Population Structure (BAPS) was used to cluster related isolates by capturing variation in the core genome. Serotype and multilocus sequence type were inferred from the genotype. Antimicrobial resistance determinants were identified from assemblies, including known macrolide resistance genes.

**Results:**

Twenty-seven BAPS clusters were assigned. These consisted of 81 sequence types (STs). Two BAPS clusters not observed in CSS-1 (*n* = 109) or CSS-2 (*n* = 69), increased in frequency in CSS-3 (*n* = 126); BAPS20 (8.73%, p 0.016) and BAPS22 (7.14%, p 0.032) but were not associated with antimicrobial resistance. Macrolide resistance within BAPS17 increased after treatment (CSS-1 *n* = 0/6, CSS-2/3 *n* = 5/5, p 0.002) and was carried on a mobile transposable element that also conferred resistance to tetracycline.

**Discussion:**

Limited changes in pneumococcal population structure were observed after the third round of MDA, suggesting treatment had little effect on the circulating lineages. An increase in macrolide resistance within one BAPS highlights the need for antimicrobial resistance surveillance in treated villages.

## Introduction

Trachoma, caused by ocular infection with *Chlamydia trachomatis*, remains the leading infectious cause of blindness worldwide. The World Health Organization recommended strategy for trachoma elimination includes mass drug administration (MDA) with the broad-spectrum antibiotic azithromycin [1,2].

Azithromycin MDA for trachoma control has been shown to have important collateral benefits [[3], [4], [5]]. In Ethiopia the mortality rate of children residing in treated villages was lower than that in untreated villages [3]. Another trial conducted in Niger, Tanzania and Malawi found biannual MDA with azithromycin significantly reduced all-cause under-5 mortality compared with placebo [5]. Substudies in that trial found an increased proportion of macrolide resistance after treatment in nasopharyngeal *Streptococcus pneumoniae* [6] and an increased frequency of macrolide resistance determinants in the gut after treatment [7]. While the mechanism underlying the reduction in mortality is still not clear, it may be partially due to the significant reduction of *S. pneumoniae* carriage [[8], [9], [10], [11]]. Other studies on azithromycin MDA for trachoma control have also found an increased risk of azithromycin-resistant pneumococcal carriage [12,13]. This has led to cautionary notes about the spread of antimicrobial resistance and requirements for adequate surveillance following MDA [14,15].

However, the effect of MDA on pneumococcal carriage is short lived with rates returning to original levels within a few months of treatment [16]. It is unclear whether the same pneumococcal strains return after treatment, or whether azithromycin MDA selects for previously suppressed strains. An Ethiopian study following intensive MDA documented decreased serotype diversity associated with a significant rise in macrolide resistance. The study concluded that clonal expansion of resistant isolates caused a decrease in the diversity of the population [17].

Bayesian Analysis of Population Structure (BAPS) is commonly used to define pneumococcal population structure in sequencing studies assessing the impact of medical inventions [[18], [19], [20]]. BAPS uses variation across the genome to cluster isolates into related groups, with increased power to detect small changes in prevalence over serotype or multilocus sequence type (MLST).

In The Gambia, we previously showed that azithromycin MDA resulted in a significant yet transient decrease in pneumococcal carriage with prevalence falling from 43% before the final round of MDA to 19% 1 month following MDA [11]. Within 6 months carriage prevalence had returned to 46% but was not associated with a significant rise in azithromycin resistance. We aimed to determine if there were changes in pneumococcal population structure following the third round of MDA with relation to azithromycin resistance.

## Methods

The Partnership for the Rapid Elimination of Trachoma (PRET) study (ClinicalTrials.gov NCT00792922) was a cluster randomized controlled trial [[21], [22], [23]]. Briefly, the study compared the effectiveness of three annual rounds versus one round of azithromycin MDA in reducing the prevalence of trachoma. Residents of a community were eligible for treatment with a single oral dose of 20 mg/kg azithromycin to a maximum of 1 g. A pneumococcal carriage study was nested within PRET [11] and was carried out in eight of the villages in the larger trial. This included two villages that had been randomized within PRET to three annual rounds of MDA (3× treatment arm) and six villages that received a single treatment round (1× treatment arm). Pneumococcal population structure was assessed by selecting isolates for sequencing from the two villages that were randomized to three annual treatment rounds. All villages had participated in a cluster (by village) randomized trial control arm of a seven-valent pneumococcal vaccine (PCV-7) between 2006 and 2008, before PCV-7 introduction in The Gambia [22]. All children under the age of 30 months when the vaccine trial started, those born during the trial period or those who moved into the study villages during the trial period were vaccinated with PCV-7.

**Fig. 1:**
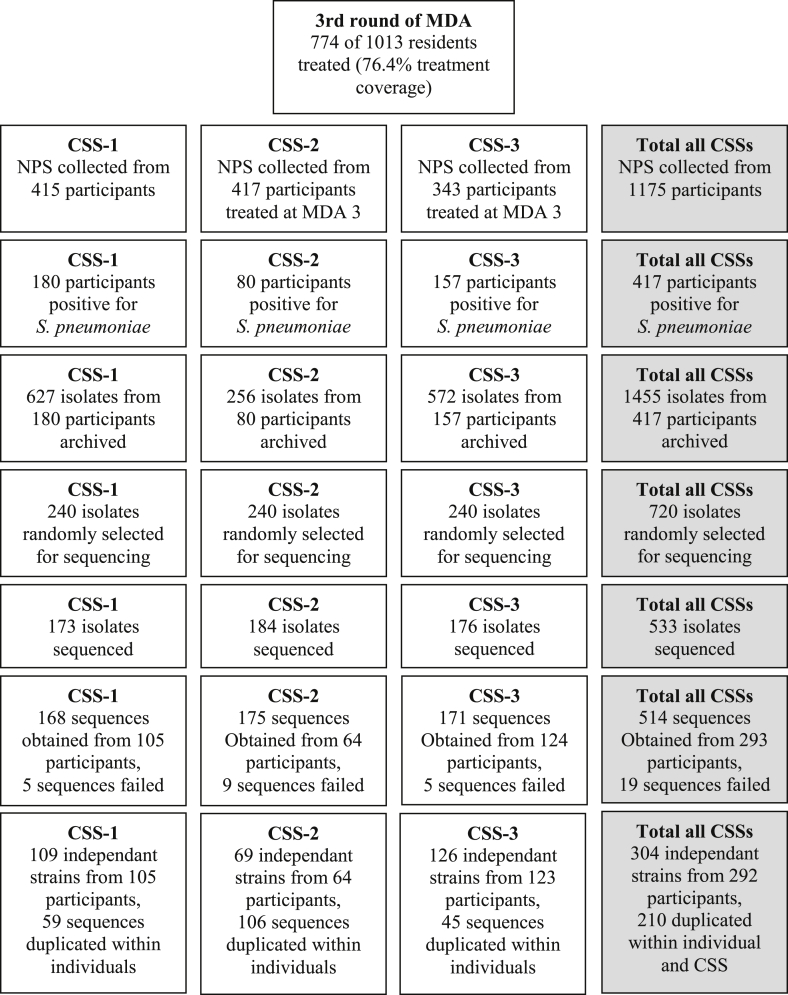
Study flow. Study flow of sampling and number of isolates available from nasopharyngeal swabs (NPS) in each cross-sectional survey (CSS) and overall.

During the pneumococcal carriage study, three cross-sectional surveys (CSSs) were conducted in the 3× treatment arm of PRET: CSS-1 (June 2010), 1 month prior to the third annual round of MDA; CSS-2 (July 2010), 1 month following the third round of MDA; and CSS-3 (December 2010), 6 months following the third round (Fig. 1), after PCV-7 was introduced into the Gambian Expanded Programme on Immunization in August 2009. Census data were gathered in the week prior to the study onset. At CSS-1, all censused children <15 years of age, present at the time of sampling were invited to participate. For individuals ≥15 years, 150 were randomly selected for participation. At CSS-2 and CSS-3, participation was restricted to those who had received azithromycin during the third round of MDA in July 2010; all censused and treated children <15 years of age and 150 randomly selected treated individuals ≥15–82 years were invited to participate. The study adhered to the tenants of the Declaration of Helsinki and was approved by The Gambia Government/Medical Research Council Unit, The Gambia Joint Ethics Committee. Written, informed consent was obtained from all participants. In the case of minors, informed consent was obtained from the parent or guardian.

Nasopharyngeal swabs (NPSs) were collected from 1175 participants. NPS samples were collected using calcium alginate swabs and inoculated into sterile vials containing skimmed-milk–tryptone–glucose glycerol transport medium. Samples were kept on wet ice, refrigerated within 8 hours of collection and moved to long-term storage at –70°C within 24 hr of collection. NPS samples were thawed at room temperature and 10 μL of medium was inoculated onto Columbia agar supplemented with 5% sheep blood and 5 μg/mL gentamicin (GBA). Inoculated plates were incubated for 18–24 hr at 35°C in 5% CO_2_. Presumptive *S. pneumoniae* colonies were identified on the basis of morphology and optochin sensitivity and up to four pneumococcal isolates representing varying colony morphologies were cultured from positive NPS [11]. In total 1455 pneumococcal isolates were archived. A random 720 isolates were selected for whole genome sequencing, restricted such that CSS-1 to 3 would be equally represented by children less than 5 years of age, between 5 and 10 years of age and by participants over 10 years of age. Isolates were screened for sensitivity to azithromycin by disc diffusion. Isolates showing intermediate or resistant phenotypes were further analysed by minimal inhibitory concentration (MIC) using the E-test (Biomerieux).

Isolates randomly selected for sequencing were revived on GBA and incubated overnight at 37°C with 5% CO_2_. A single colony was inoculated into 20 mL of Todd–Hewitt broth containing 0.5% yeast extract and re-incubated overnight. The overnight broth cultures were pelleted by centrifugation and DNA was extracted using the Wizard Genomic DNA Purification Kit (Promega Corporation, Madison, WI, USA). DNA quantification was performed using PicoGreen staining (Invitrogen, Paisley, UK). DNA extracts from 533 samples that could be revived and that yielded at least 500 ng of DNA were shipped, on dry ice, to the Wellcome Sanger Institute for sequencing.

DNA was sequenced on Illumina Hiseq platforms using pair-end read lengths of 100 base pairs. Data for 514 sequenced isolates were not heavily contaminated, had sufficient coverage to be analysed and were confirmed as pneumococci. These samples represented 293 participants, of which 75% were children aged 0–9 years. Core genes were defined using Roary [24]. The core alignment was clustered using hierBAPS [25] to determine BAPS clusters, with a second level of hierarchical clustering to determine BAPS subclusters. The core alignment was reduced to single nucleotide polymorphism (SNP) sites [26] and used to produce a midpoint rooted RAxML phylogeny [27]. SNPs were reconstructed on the tree using the acctran parsimony method. Serotypes were inferred from the genomic data [28]. Antimicrobial resistance identification by assembly (ARIBA) [29] and a pneumococcal typing tool [30] were used to screen isolates for known resistance conferring genes and mutations. Metadata for 514 isolates with European Nucleotide Archive (ENA) accession numbers are available (please see supplementary material) and paired with the phylogenetic tree (https://microreact.org/project/MDA_pneumo).

Only one representative of each sequence type (ST) from the same individual and timepoint was included in the statistical analysis as these represented duplicated sampling from multiple colonies from an individual NPS, leaving 304 independent isolates of the 514 appropriate to include in statistical analysis. The diversity of serotypes and STs was assessed using Simpson's Diversity index, where 1 represents infinite diversity and 0 no diversity.

The proportion for each serotype/genotype within each CSS was calculated. Confidence intervals were calculated at 95% using the exact method of biconf in the Hmisc package, R v3.4.1 [31]. The two-tailed Fisher exact test was used to detect significant changes in the proportion of each serotype/genotype between two time-points when at least five isolates represented the serotype/genotype. p Values were adjusted for multiple testing using the ‘BH’ method of p.adjust in the stats package, R v3.4.1 [32]. The 304 independent isolates provided 80% power with 95% confidence to detect a fivefold increase in a BAPS cluster between CSS-1 and CSS-2 and fourfold increase between CCS-1 and CSS-3.

## Results

During the initial carriage study, 1455 isolates were recovered from participants in the 3× treatment arm. Of these, 720 were randomly selected for sequencing. In total 533 of these were successfully revived from glycerol stocks and yielded at least 500 ng of DNA. Quality sequence data were obtained for 514 isolates. After excluding multiple instances of a ST from the same participant and time point, 304 independent isolates were available to assess temporal distribution and diversity (Fig. 1).

### Population structure

**Fig. 2:**
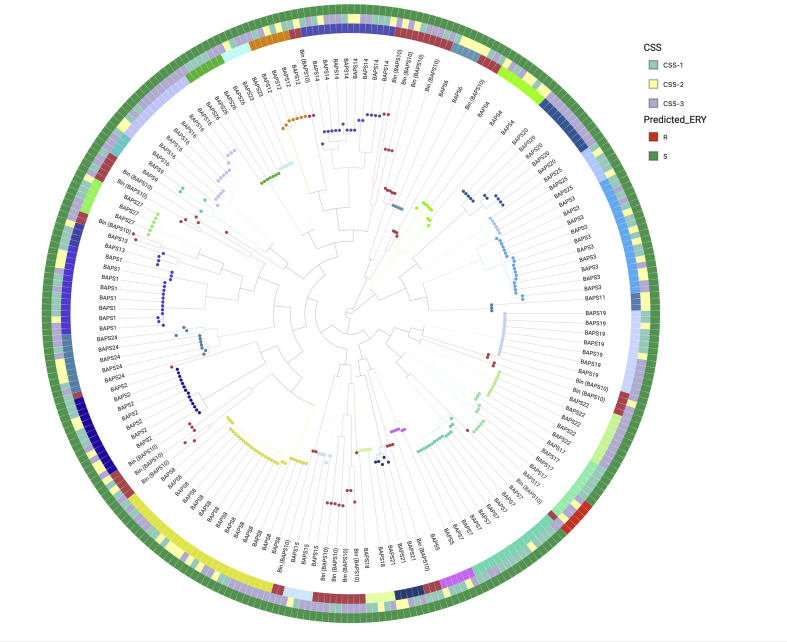
Distribution of isolates predicted to be resistant to erythromycin across BAPS clusters and CSS. Phylogenetic tree of single nucleotide polymorphisms in the core genes reconstructed on the tree of the 304 randomly sampled isolates. Scale is in SNPs. The nodes and innermost ring represent the first level BAPS clusters which are annotated, of which BAPS10 (red) is the bin cluster representing un-clustered isolates spread across the tree. BAPS cluster nomenclature represents the largest (BAPS1) through to the smallest cluster (BAPS27). The middle ring depicts the CSS in which the isolate was collected CSS-1(green), CSS-2 (yellow) CSS-3 (purple). The outermost ring depicts each isolate's predicted susceptibility to erythromycin across the tree, resistant (red), sensitive (green). A fully interactive view of this figure is available at https://microreact.org/project/MDA_pneumo/9861bdea.

BAPS revealed 27 BAPS clusters (Fig. 2). We did not observe significant changes in the proportion of isolates in any BAPS cluster between CSS-1 and CSS-2 (Table 1). A significant increase between CSS-1 and CSS-3 was observed for BAPS20 (p 0.016) and BAPS22 (p 0.032). Neither BAPS cluster was observed at CSS-1 or CSS-2 (Table 1) and comprised exclusively serotype 16F (ST8949/13858) and 45 (ST2831), respectively, in CSS-3. A second round of BAPS clustering identified 81 BAPS subclusters. None was observed to significantly change in proportion (Table S1). We observed 36 serotypes and 81 STs. No serotype or ST was found to vary significantly in their proportion of the population (Tables S2 and S3). At each CSS, serotype and ST diversity was high. No decrease in diversity was seen following the round of MDA (Table 2).

**Table 1:** Proportion of isolates belonging to BAPS clusters at each CSS

BAPS clusters	CSS-1	CSS-2	CSS-1 vs CSS-2	CSS-3	CSS-1 vs CSS-3
	Proportion (*n* = 109)	Proportion (*n* = 69)	p value	Proportion (*n* = 126)	p value
	% (95% CI)	% (95% CI)	(direction)	% (95% CI)	(direction)
1	6.42 (2.62, 12.78)	5.8 (1.6, 14.18)	1.000	3.17 (0.87, 7.93)	0.599
2	5.5 (2.05, 11.6)	7.25 (2.39, 16.11)	1.000	2.38 (0.49, 6.8)	0.599
3	8.26 (3.85, 15.1)	8.7 (3.26, 17.97)	1.000	3.97 (1.3, 9.02)	0.481
4	1.83 (0.22, 6.47)	7.25 (2.39, 16.11)	0.552	1.59 (0.19, 5.62)	NA
5	2.75 (0.57, 7.83)	2.9 (0.35, 10.08)	1.000	0.79 (0.02, 4.34)	NA
6	0.92 (0.02, 5.01)	5.8 (1.6, 14.18)	0.552	0 (0, 2.89)	NA
7	11.01 (5.82, 18.44)	1.45 (0.04, 7.81)	0.262	2.38 (0.49, 6.8)	0.071
8	7.34 (3.22, 13.95)	10.14 (4.18, 19.79)	1.000	11.11 (6.21, 17.94)	0.599
9	0.92 (0.02, 5.01)	0 (0, 5.21)	NA	2.38 (0.49, 6.8)	NA
10^a^	13.76 (7.91, 21.68)	10.14 (4.18, 19.79)	1.000	20.63 (13.94, 28.75)	0.481
11	0 (0, 3.33)	4.35 (0.91, 12.18)	NA	0 (0, 2.89)	NA
12	2.75 (0.57, 7.83)	2.9 (0.35, 10.08)	1.000	1.59 (0.19, 5.62)	0.808
13	2.75 (0.57, 7.83)	1.45 (0.04, 7.81)	NA	0 (0, 2.89)	NA
14	5.5 (2.05, 11.6)	5.8 (1.6, 14.18)	1.000	4.76 (1.77, 10.08)	1.000
15	0.92 (0.02, 5.01)	1.45 (0.04, 7.81)	NA	2.38 (0.49, 6.8)	NA
16	1.83 (0.22, 6.47)	2.9 (0.35, 10.08)	NA	7.94 (3.87, 14.11)	0.158
17	5.5 (2.05, 11.6)	2.9 (0.35, 10.08)	1.000	2.38 (0.49, 6.8)	0.599
18	2.75 (0.57, 7.83)	1.45 (0.04, 7.81)	NA	0.79 (0.02, 4.34)	NA
19	5.5 (2.05, 11.6)	2.9 (0.35, 10.08)	1.000	4.76 (1.77, 10.08)	1.000
20	0 (0, 3.33)	0 (0, 5.21)	NA	8.73 (4.44, 15.08)	0.016 (↑)
21	0.92 (0.02, 5.01)	2.9 (0.35, 10.08)	NA	1.59 (0.19, 5.62)	NA
22	0 (0, 3.33)	0 (0, 5.21)	NA	7.14 (3.32, 13.13)	0.032 (↑)
23	0.92 (0.02, 5.01)	1.45 (0.04, 7.81)	NA	2.38 (0.49, 6.8)	NA
24	1.83 (0.22, 6.47)	5.8 (1.6, 14.18)	0.782	3.17 (0.87, 7.93)	0.808
25	3.67 (1.01, 9.13)	2.9 (0.35, 10.08)	1.00	0 (0, 2.89)	NA
26	3.67 (1.01, 9.13)	0 (0, 5.21)	NA	2.38 (0.49, 6.8)	0.808
27	2.75 (0.57, 7.83)	1.45 (0.04, 7.81)	NA	1.59 (0.19, 5.62)	0.808

NA, <5 isolates, not tested.

a bin cluster.

**Table 2:** Serotype and ST diversity at each CSS

	CSS-1	CSS-2	CSS-3
Serotype^a^	0.94	0.93	0.94
ST^a^	0.96	0.96	0.96

a Diversity was assessed using Simpsons Diversity index (Dominance Index Approximation), which represents the probability that two randomly selected strains are the same, where 1 means they are different and 0 indicates they are the same.

### Antimicrobial resistance

No isolates carried macrolide resistance genes in CSS-1 (*n* = 109), 2/69 were observed in CSS-2 and 4/126 in CSS-3; this represented a non-significant increase (p 1). However, 5/6 were serotype 20, ST1794 strains of BAPS17. They carried the macrolide efflux genes *mef*(A) and *msr*(D) [33] (Fig. 2). These were carried in a Tn2010 element, which also carried the tetracycline resistance gene *tet*(M) [23]. The isolates also had known co-trimoxazole resistance mutations in FolA and FolP. The proportion of BAPS17 isolates predicted to be erythromycin, tetracycline and co-trimoxazole resistant within BAPS17 increased significantly between CSS-1 (zero of six BAPS17 isolates) and CSS-2/3 (five of five BAPS17 isolates, p 0.002). Only one additional isolate, belonging to serotype 13, carried the macrolide efflux gene *mef*(E) [22].

## Discussion

MDA with azithromycin for trachoma elimination in The Gambia was associated with a short-term decrease in *S. pneumoniae* carriage [11]. To evaluate whether communities were repopulated with the same strains following treatment or whether MDA gave rise to previously suppressed clones, we compared pneumococcal isolates sampled from communities before and after a third round of MDA. Our results show little change in population structure following a round of MDA in communities where the number of circulating lineages was high and the prevalence of macrolide resistance low.

The number of BAPS clusters we identified was higher than other published carriage datasets using this approach, likely reflecting the broader pneumococcal diversity in The Gambia with small numbers representing numerous circulating lineages [19,20]. No changes in BAPS clusters were identified at CSS-2, suggesting any change that MDA had on the population structure was short lived, or that these changes were below our detection limit. Greater variation was seen between CSS-1 and CSS-3, 6 months following MDA. These changes could be a result of natural temporal fluctuations [34] or seasonal change [35]; CSS-1 and CCS-2 were conducted in the wet season while CSS-3 was carried out in the dry season. Only BAPS20 and BAPS22, showed an increase between CSS-1 and CSS-3. However, these changes were not associated with macrolide resistance and are difficult to attribute to the MDA.

Macrolide resistance conferred by *mef*(A) was confined to BAPS17; this lineage did not increase significantly following treatment. However, the proportion of macrolide resistance isolates within BAPS17 was higher at both CSS-2 and CSS-3, indicating an increase after MDA. No macrolide resistance was observed in CSS-1 in any BAPS cluster. We cannot rule out low-level presence of macrolide-resistant BAPS17 strains circulating in the community at CSS-1 due to the relatively small sample size in the background of high diversity. BAPS17 ST1794 was only observed once in a published international dataset [36] despite inclusion of 1249 carriage isolates from The Gambia. BAPS17 isolates belong to the Global Pneumococcal Sequence Cluster GPSC61 within which *mef*(A) and/or *erm*(B) were not detected, suggesting GPSC61 is not typically associated with macrolide resistance. Although the numbers are small, all isolates that carried macrolide resistance genes concurrently carried the tetracycline resistance gene *tet*(M) in a mobile genetic element. If MDA selects for strains carrying mobile genetic elements, often encoding multiple antibiotic resistance genes, there is potential for increases in multidrug resistance [37]. Macrolide resistance was at undetectable levels in this study before the intervention, in other settings with established macrolide resistance in pneumococci, the potential for rises in macrolide-resistant pneumococci after MDA with azithromycin may be greater. Further studies with larger sample sizes would be needed to adequately address this concern and would be important if azithromycin MDA were to be considered as an intervention to reduce child mortality [5].

Only 75% of randomly selected pneumococcal isolates from the carriage survey could be sequenced, due to insufficient growth. This may have resulted in an underrepresentation of serotypes or STs that are slow growing or otherwise less robust. The sample size relative to the high pneumococcal diversity observed in our study limited our ability to detect smaller changes in serotype and genotype frequency following MDA. We did not have isolates obtained before MDA in this study. We therefore cannot rule out that the 3-year MDA programme, as a whole, affected pneumococcal population structure, although it did not lead to a substantial increase in macrolide resistance as only 6/304 (2%) carried macrolide resistance genes. We only screened for known genetic determinants of macrolide resistance, however, *mef* genes and/or *erm*(B) have been reported to have a sensitivity of 97% and specificity of 99% for phenotypic resistance in pneumococci [36]. Finally, the original pneumococcal carriage study was conducted in villages where the majority of children under the age of 6 years had received PCV-7 vaccination. As we do not hold vaccination data on individual study participants, PCV-7 vaccination may have impacted the results of the current study. It is known that PCVs do not modify carriage rates, but altered the serotype distribution by increasing non-vaccine serotypes [37,38]. Therefore, our findings should be generalizable to other countries where MDA for trachoma control and PCV vaccination are both a part of the public health strategy.

In this study we observed few changes in pneumococcal population structure. A small number of isolates had macrolide resistance genes, but these were found on a mobile genetic element carrying other antimicrobial resistance genes in MDR strains. Further studies with larger sample sizes would be needed to determine whether azithromycin MDA selects for MDR.

## Transparency declaration

This work was funded by grants from the 10.13039/100000865Bill and Melinda Gates Foundation (grant number 48027) (S.E.B., J.H., E.H.E.), 10.13039/100010269Wellcome Trust Programme Grants (GR079246MA and WT093368MA) (M.H., R.L.B., D.C.W.M.) and by core funds of the 10.13039/100010269Wellcome Trust Sanger Institute (R.G., S.D.B.) and the MRC Unit The Gambia at LSHTM (E.B., A.R.). The funders had no part in the study design; in the collection, analysis and interpretation of data; in the writing of the report; and in the decision to submit the paper for publication. A.S. was supported by the Gambian MoH, D.C.W.M. and R.L.B. also received LSHTM institute support. The authors have nothing to disclose.
