# Supplementary material for: Mass drug administration with azithromycin for trachoma elimination and the population structure of Streptococcus pneumoniae in the nasopharynx
Source: Clin Microbiol Infect. 2021 Jun;27(6):864–70. doi: 10.1016/j.cmi.2020.07.039 (PMC8203556; doi:10.1016/j.cmi.2020.07.039)
Supplement: Multimedia component 1 [file mmc1.docx]

Supplementary Table 1. Proportion of isolates belonging to BAPS sub-clusters at each CSS.

| BAPS  sub-cluster | CSS-1 | CSS-2 | CSS-1 vs CSS-2 | CSS-3 | CSS-1 vs CSS-3 |
| --- | --- | --- | --- | --- | --- |
|  | Proportion (N=109) | Proportion (N=69) | p value | Proportion (N=126) | p value |
|  | % (95% CI) | % (95% CI) |  | % (95% CI) |  |
| 1 | 5.5 (2.05, 11.6) | 2.9 (0.35, 10.08) | 0.733 | 3.17 (0.87, 7.93) | 0.669 |
| 2 | 0.92 (0.02, 5.01) | 2.9 (0.35, 10.08) | NA | 0 (0, 2.89) | NA |
| 3 | 5.5 (2.05, 11.6) | 2.9 (0.35, 10.08) | 0.733 | 0 (0, 2.89) | 0.055 |
| 4 | 0 (0, 3.33) | 0 (0, 5.21) | NA | 0.79 (0.02, 4.34) | NA |
| 5 | 0 (0, 3.33) | 4.35 (0.91, 12.18) | NA | 1.59 (0.19, 5.62) | NA |
| 6 | 1.83 (0.22, 6.47) | 0 (0, 5.21) | NA | 0 (0, 2.89) | NA |
| 7 | 0.92 (0.02, 5.01) | 4.35 (0.91, 12.18) | NA | 2.38 (0.49, 6.8) | NA |
| 8 | 2.75 (0.57, 7.83) | 1.45 (0.04, 7.81) | NA | 1.59 (0.19, 5.62) | 0.748 |
| 9 | 1.83 (0.22, 6.47) | 2.9 (0.35, 10.08) | NA | 0 (0, 2.89) | NA |
| 10 | 0.92 (0.02, 5.01) | 0 (0, 5.21) | NA | 0 (0, 2.89) | NA |
| 11 | 0.92 (0.02, 5.01) | 4.35 (0.91, 12.18) | NA | 1.59 (0.19, 5.62) | NA |
| 12 | 0.92 (0.02, 5.01) | 2.9 (0.35, 10.08) | NA | 0 (0, 2.89) | NA |
| 13 | 0 (0, 3.33) | 1.45 (0.04, 7.81) | NA | 0.79 (0.02, 4.34) | NA |
| 14 | 2.75 (0.57, 7.83) | 1.45 (0.04, 7.81) | NA | 0 (0, 2.89) | NA |
| 15 | 0.92 (0.02, 5.01) | 1.45 (0.04, 7.81) | NA | 0 (0, 2.89) | NA |
| 16 | 0 (0, 3.33) | 4.35 (0.91, 12.18) | NA | 0 (0, 2.89) | NA |
| 17 | 0 (0, 3.33) | 0 (0, 5.21) | NA | 0 (0, 2.89) | NA |
| 18 | 5.5 (2.05, 11.6) | 1.45 (0.04, 7.81) | 0.733 | 0 (0, 2.89) | 0.055 |
| 19 | 3.67 (1.01, 9.13) | 0 (0, 5.21) | NA | 1.59 (0.19, 5.62) | 0.630 |
| 20 | 0 (0, 3.33) | 0 (0, 5.21) | NA | 0.79 (0.02, 4.34) | NA |
| 21 | 1.83 (0.22, 6.47) | 0 (0, 5.21) | NA | 0 (0, 2.89) | NA |
| 22 | 4.59 (1.51, 10.38) | 10.14 (4.18, 19.79) | 0.733 | 2.38 (0.49, 6.8) | 0.660 |
| 23 | 0 (0, 3.33) | 0 (0, 5.21) | NA | 4.76 (1.77, 10.08) | 0.095 |
| 24 | 2.75 (0.57, 7.83) | 0 (0, 5.21) | NA | 1.59 (0.19, 5.62) | 0.748 |
| 25 | 0 (0, 3.33) | 0 (0, 5.21) | NA | 2.38 (0.49, 6.8) | NA |
| 26 | 0 (0, 3.33) | 0 (0, 5.21) | NA | 1.59 (0.19, 5.62) | NA |
| 27 | 0 (0, 3.33) | 0 (0, 5.21) | NA | 0.79 (0.02, 4.34) | NA |
| 28 | 0.92 (0.02, 5.01) | 0 (0, 5.21) | NA | 0 (0, 2.89) | NA |
| 29 | 1.83 (0.22, 6.47) | 1.45 (0.04, 7.81) | NA | 0.79 (0.02, 4.34) | NA |
| 30 | 0 (0, 3.33) | 0 (0, 5.21) | NA | 1.59 (0.19, 5.62) | NA |
| 31 | 0.92 (0.02, 5.01) | 0 (0, 5.21) | NA | 3.17 (0.87, 7.93) | 0.630 |
| 32 | 3.67 (1.01, 9.13) | 1.45 (0.04, 7.81) | 0.754 | 4.76 (1.77, 10.08) | 0.800 |
| 33 | 2.75 (0.57, 7.83) | 0 (0, 5.21) | NA | 0 (0, 2.89) | NA |
| 34^1^ | 4.59 (1.51, 10.38) | 7.25 (2.39, 16.11) | 0.733 | 10.32 (5.61, 17) | 0.279 |
| 35 | 0 (0, 3.33) | 1.45 (0.04, 7.81) | NA | 0 (0, 2.89) | NA |
| 36 | 0 (0, 3.33) | 1.45 (0.04, 7.81) | NA | 0 (0, 2.89) | NA |
| 37 | 0 (0, 3.33) | 1.45 (0.04, 7.81) | NA | 0 (0, 2.89) | NA |
| 38 | 0.92 (0.02, 5.01) | 0 (0, 5.21) | NA | 0.79 (0.02, 4.34) | NA |
| 39 | 1.83 (0.22, 6.47) | 2.9 (0.35, 10.08) | NA | 0.79 (0.02, 4.34) | NA |
| 40 | 2.75 (0.57, 7.83) | 0 (0, 5.21) | NA | 0 (0, 2.89) | NA |
| 41 | 0 (0, 3.33) | 1.45 (0.04, 7.81) | NA | 0 (0, 2.89) | NA |
| 42 | 2.75 (0.57, 7.83) | 4.35 (0.91, 12.18) | 0.754 | 3.17 (0.87, 7.93) | 1.000 |
| 43 | 0 (0, 3.33) | 0 (0, 5.21) | NA | 1.59 (0.19, 5.62) | NA |
| 44 | 2.75 (0.57, 7.83) | 1.45 (0.04, 7.81) | NA | 0 (0, 2.89) | NA |
| 46 | 0.92 (0.02, 5.01) | 1.45 (0.04, 7.81) | NA | 0.79 (0.02, 4.34) | NA |
| 47 | 0 (0, 3.33) | 0 (0, 5.21) | NA | 0.79 (0.02, 4.34) | NA |
| 48 | 0 (0, 3.33) | 0 (0, 5.21) | NA | 0.79 (0.02, 4.34) | NA |
| 49 | 0 (0, 3.33) | 0 (0, 5.21) | NA | 3.97 (1.3, 9.02) | 0.142 |
| 50 | 1.83 (0.22, 6.47) | 1.45 (0.04, 7.81) | NA | 0.79 (0.02, 4.34) | NA |
| 51 | 0 (0, 3.33) | 1.45 (0.04, 7.81) | NA | 0 (0, 2.89) | NA |
| 52 | 0 (0, 3.33) | 0 (0, 5.21) | NA | 3.17 (0.87, 7.93) | NA |
| 53 | 0 (0, 3.33) | 2.9 (0.35, 10.08) | NA | 2.38 (0.49, 6.8) | NA |
| 54 | 2.75 (0.57, 7.83) | 0 (0, 5.21) | NA | 0 (0, 2.89) | NA |
| 55 | 2.75 (0.57, 7.83) | 0 (0, 5.21) | NA | 0 (0, 2.89) | NA |
| 56 | 0.92 (0.02, 5.01) | 0 (0, 5.21) | NA | 0.79 (0.02, 4.34) | NA |
| 57 | 0.92 (0.02, 5.01) | 0 (0, 5.21) | NA | 0 (0, 2.89) | NA |
| 58 | 0.92 (0.02, 5.01) | 1.45 (0.04, 7.81) | NA | 0 (0, 2.89) | NA |
| 59 | 5.5 (2.05, 11.6) | 2.9 (0.35, 10.08) | 0.733 | 0 (0, 2.89) | 0.055 |
| 60 | 0 (0, 3.33) | 0 (0, 5.21) | NA | 4.76 (1.77, 10.08) | 0.095 |
| 61 | 0 (0, 3.33) | 0 (0, 5.21) | NA | 3.97 (1.3, 9.02) | 0.142 |
| 62 | 0 (0, 3.33) | 0 (0, 5.21) | NA | 1.59 (0.19, 5.62) | NA |
| 63 | 0 (0, 3.33) | 0 (0, 5.21) | NA | 3.17 (0.87, 7.93) | NA |
| 64 | 0.92 (0.02, 5.01) | 0 (0, 5.21) | NA | 0 (0, 2.89) | NA |
| 65 | 0 (0, 3.33) | 2.9 (0.35, 10.08) | NA | 0 (0, 2.89) | NA |
| 66 | 0 (0, 3.33) | 0 (0, 5.21) | NA | 0.79 (0.02, 4.34) | NA |
| 67 | 0 (0, 3.33) | 0 (0, 5.21) | NA | 0.79 (0.02, 4.34) | NA |
| 68 | 0 (0, 3.33) | 0 (0, 5.21) | NA | 5.56 (2.26, 11.11) | 0.072 |
| 69 | 0 (0, 3.33) | 0 (0, 5.21) | NA | 1.59 (0.19, 5.62) | NA |
| 70 | 0 (0, 3.33) | 0 (0, 5.21) | NA | 1.59 (0.19, 5.62) | NA |
| 71 | 0.92 (0.02, 5.01) | 1.45 (0.04, 7.81) | NA | 0 (0, 2.89) | NA |
| 72 | 0 (0, 3.33) | 0 (0, 5.21) | NA | 0.79 (0.02, 4.34) | NA |
| 73 | 0.92 (0.02, 5.01) | 5.8 (1.6, 14.18) | 0.733 | 1.59 (0.19, 5.62) | NA |
| 74 | 0.92 (0.02, 5.01) | 0 (0, 5.21) | NA | 0 (0, 2.89) | NA |
| 75 | 0 (0, 3.33) | 0 (0, 5.21) | NA | 1.59 (0.19, 5.62) | NA |
| 76 | 2.75 (0.57, 7.83) | 2.9 (0.35, 10.08) | 1.000 | 0 (0, 2.89) | NA |
| 77 | 0.92 (0.02, 5.01) | 0 (0, 5.21) | NA | 0 (0, 2.89) | NA |
| 78 | 3.67 (1.01, 9.13) | 0 (0, 5.21) | NA | 1.59 (0.19, 5.62) | 0.630 |
| 79 | 0 (0, 3.33) | 0 (0, 5.21) | NA | 0.79 (0.02, 4.34) | NA |
| 80 | 2.75 (0.57, 7.83) | 1.45 (0.04, 7.81) | NA | 0 (0, 2.89) | NA |
| 81 | 0 (0, 3.33) | 0 (0, 5.21) | NA | 1.59 (0.19, 5.62) | NA |

^1^bin cluster

NA =<5 isolates, not tested

Supplementary Table 2. Proportion of isolates belonging to a particular ST at each CSS.

| ST | CSS-1 | CSS-2 | CSS-1 vs CSS-2 | CSS-3 | CSS-1 vs CSS-3 |
| --- | --- | --- | --- | --- | --- |
|  | Proportion (N=109) | Proportion (N=69) | p value | Proportion (N=126) | p value |
|  | % (95% CI) | % (95% CI) |  | % (95% CI) |  |
| 63 | 0.92 (0.02, 5.01) | 0 (0, 5.21) | NA | 1.59 (0.19, 5.62) | NA |
| 177 | 0.92 (0.02, 5.01) | 0 (0, 5.21) | NA | 0 (0, 2.89) | NA |
| 180 | 0 (0, 3.33) | 0 (0, 5.21) | NA | 2.38 (0.49, 6.8) | NA |
| 260 | 0 (0, 3.33) | 0 (0, 5.21) | NA | 0.79 (0.02, 4.34) | NA |
| 361 | 2.75 (0.57, 7.83) | 1.45 (0.04, 7.81) | NA | 0 (0, 2.89) | NA |
| 447 | 0 (0, 3.33) | 0 (0, 5.21) | NA | 1.59 (0.19, 5.62) | NA |
| 458 | 2.75 (0.57, 7.83) | 0 (0, 5.21) | NA | 0 (0, 2.89) | NA |
| 802 | 0.92 (0.02, 5.01) | 1.45 (0.04, 7.81) | NA | 2.38 (0.49, 6.8) | NA |
| 847 | 5.5 (2.05, 11.6) | 7.25 (2.39, 16.11) | 1.000 | 2.38 (0.49, 6.8) | 0.653 |
| 910 | 5.5 (2.05, 11.6) | 2.9 (0.35, 10.08) | 0.974 | 3.17 (0.87, 7.93) | 0.728 |
| 913 | 0.92 (0.02, 5.01) | 5.8 (1.6, 14.18) | 0.375 | 1.59 (0.19, 5.62) | NA |
| 915 | 2.75 (0.57, 7.83) | 1.45 (0.04, 7.81) | NA | 1.59 (0.19, 5.62) | 0.776 |
| 925 | 0.92 (0.02, 5.01) | 1.45 (0.04, 7.81) | NA | 0 (0, 2.89) | NA |
| 989 | 0 (0, 3.33) | 1.45 (0.04, 7.81) | NA | 0 (0, 2.89) | NA |
| 1469 | 2.75 (0.57, 7.83) | 2.9 (0.35, 10.08) | 1.000 | 0 (0, 2.89) | NA |
| 1735 | 0 (0, 3.33) | 0 (0, 5.21) | NA | 0.79 (0.02, 4.34) | NA |
| 1736 | 0.92 (0.02, 5.01) | 0 (0, 5.21) | NA | 0 (0, 2.89) | NA |
| 1737 | 0 (0, 3.33) | 0 (0, 5.21) | NA | 1.59 (0.19, 5.62) | NA |
| 1778 | 3.67 (1.01, 9.13) | 0 (0, 5.21) | NA | 1.59 (0.19, 5.62) | 0.653 |
| 1794 | 0 (0, 3.33) | 2.9 (0.35, 10.08) | NA | 2.38 (0.49, 6.8) | NA |
| 2169 | 0 (0, 3.33) | 0 (0, 5.21) | NA | 0.79 (0.02, 4.34) | NA |
| 2174 | 0 (0, 3.33) | 0 (0, 5.21) | NA | 1.59 (0.19, 5.62) | NA |
| 2178 | 0.92 (0.02, 5.01) | 1.45 (0.04, 7.81) | NA | 0.79 (0.02, 4.34) | NA |
| 2831 | 0 (0, 3.33) | 0 (0, 5.21) | NA | 7.14 (3.32, 13.13) | 0.056 |
| 3310 | 0 (0, 3.33) | 0 (0, 5.21) | NA | 0.79 (0.02, 4.34) | NA |
| 3329 | 1.83 (0.22, 6.47) | 2.9 (0.35, 10.08) | NA | 4.76 (1.77, 10.08) | 0.653 |
| 3404 | 0.92 (0.02, 5.01) | 2.9 (0.35, 10.08) | NA | 0 (0, 2.89) | NA |
| 3406 | 0 (0, 3.33) | 0 (0, 5.21) | NA | 1.59 (0.19, 5.62) | NA |
| 3407 | 8.26 (3.85, 15.1) | 8.7 (3.26, 17.97) | 1.000 | 2.38 (0.49, 6.8) | 0.248 |
| 4033 | 7.34 (3.22, 13.95) | 8.7 (3.26, 17.97) | 1.000 | 11.11 (6.21, 17.94) | 0.653 |
| 4832 | 0 (0, 3.33) | 0 (0, 5.21) | NA | 0.79 (0.02, 4.34) | NA |
| 4989 | 0 (0, 3.33) | 1.45 (0.04, 7.81) | NA | 0.79 (0.02, 4.34) | NA |
| 5103 | 0.92 (0.02, 5.01) | 2.9 (0.35, 10.08) | NA | 0 (0, 2.89) | NA |
| 5349 | 0.92 (0.02, 5.01) | 1.45 (0.04, 7.81) | NA | 2.38 (0.49, 6.8) | NA |
| 5505 | 2.75 (0.57, 7.83) | 1.45 (0.04, 7.81) | NA | 0.79 (0.02, 4.34) | NA |
| 5511 | 0 (0, 3.33) | 0 (0, 5.21) | NA | 0.79 (0.02, 4.34) | NA |
| 5515 | 0.92 (0.02, 5.01) | 0 (0, 5.21) | NA | 0 (0, 2.89) | NA |
| 5516 | 0 (0, 3.33) | 1.45 (0.04, 7.81) | NA | 0 (0, 2.89) | NA |
| 5521 | 5.5 (2.05, 11.6) | 2.9 (0.35, 10.08) | 0.974 | 4.76 (1.77, 10.08) | 1.000 |
| 5702 | 2.75 (0.57, 7.83) | 2.9 (0.35, 10.08) | 1.000 | 1.59 (0.19, 5.62) | 0.776 |
| 5706 | 0.92 (0.02, 5.01) | 0 (0, 5.21) | NA | 0 (0, 2.89) | NA |
| 5719 | 3.67 (1.01, 9.13) | 0 (0, 5.21) | NA | 1.59 (0.19, 5.62) | 0.653 |
| 5722 | 0 (0, 3.33) | 1.45 (0.04, 7.81) | NA | 0 (0, 2.89) | NA |
| 5726 | 1.83 (0.22, 6.47) | 0 (0, 5.21) | NA | 0 (0, 2.89) | NA |
| 5734 | 0.92 (0.02, 5.01) | 4.35 (0.91, 12.18) | NA | 1.59 (0.19, 5.62) | NA |
| 5823 | 0 (0, 3.33) | 0 (0, 5.21) | NA | 0.79 (0.02, 4.34) | NA |
| 5902 | 2.75 (0.57, 7.83) | 0 (0, 5.21) | NA | 0 (0, 2.89) | NA |
| 7336 | 0 (0, 3.33) | 1.45 (0.04, 7.81) | NA | 0.79 (0.02, 4.34) | NA |
| 8949 | 0 (0, 3.33) | 0 (0, 5.21) | NA | 5.56 (2.26, 11.11) | 0.074 |
| 10542 | 0 (0, 3.33) | 0 (0, 5.21) | NA | 1.59 (0.19, 5.62) | NA |
| 10593 | 2.75 (0.57, 7.83) | 0 (0, 5.21) | NA | 0 (0, 2.89) | NA |
| 10607 | 0 (0, 3.33) | 2.9 (0.35, 10.08) | NA | 0 (0, 2.89) | NA |
| 10610 | 0 (0, 3.33) | 0 (0, 5.21) | NA | 1.59 (0.19, 5.62) | NA |
| 10679 | 5.5 (2.05, 11.6) | 1.45 (0.04, 7.81) | 0.835 | 0 (0, 2.89) | 0.065 |
| 10777 | 0 (0, 3.33) | 0 (0, 5.21) | NA | 3.17 (0.87, 7.93) | NA |
| 10963 | 1.83 (0.22, 6.47) | 0 (0, 5.21) | NA | 0 (0, 2.89) | NA |
| 11707 | 0 (0, 3.33) | 0 (0, 5.21) | NA | 0.79 (0.02, 4.34) | NA |
| 11710 | 0 (0, 3.33) | 0 (0, 5.21) | NA | 0.79 (0.02, 4.34) | NA |
| 11720 | 0.92 (0.02, 5.01) | 5.8 (1.6, 14.18) | 0.375 | 0 (0, 2.89) | NA |
| 11756 | 0.92 (0.02, 5.01) | 0 (0, 5.21) | NA | 0 (0, 2.89) | NA |
| 11758 | 0 (0, 3.33) | 0 (0, 5.21) | NA | 0.79 (0.02, 4.34) | NA |
| 12287 | 2.75 (0.57, 7.83) | 1.45 (0.04, 7.81) | NA | 0 (0, 2.89) | NA |
| 12288 | 0 (0, 3.33) | 4.35 (0.91, 12.18) | NA | 0 (0, 2.89) | NA |
| 12314 | 0 (0, 3.33) | 0 (0, 5.21) | NA | 2.38 (0.49, 6.8) | NA |
| 12692 | 0.92 (0.02, 5.01) | 0 (0, 5.21) | NA | 0.79 (0.02, 4.34) | NA |
| 13845 | 0 (0, 3.33) | 0 (0, 5.21) | NA | 0.79 (0.02, 4.34) | NA |
| 13846 | 0.92 (0.02, 5.01) | 0 (0, 5.21) | NA | 0 (0, 2.89) | NA |
| 13847 | 0.92 (0.02, 5.01) | 1.45 (0.04, 7.81) | NA | 0 (0, 2.89) | NA |
| 13848 | 0 (0, 3.33) | 0 (0, 5.21) | NA | 1.59 (0.19, 5.62) | NA |
| 13849 | 0.92 (0.02, 5.01) | 0 (0, 5.21) | NA | 0 (0, 2.89) | NA |
| 13850 | 0.92 (0.02, 5.01) | 0 (0, 5.21) | NA | 0 (0, 2.89) | NA |
| 13850 | 0.92 (0.02, 5.01) | 0 (0, 5.21) | NA | 0 (0, 2.89) | NA |
| 13852 | 0 (0, 3.33) | 0 (0, 5.21) | NA | 1.59 (0.19, 5.62) | NA |
| 13853 | 1.83 (0.22, 6.47) | 1.45 (0.04, 7.81) | NA | 2.38 (0.49, 6.8) | 1.000 |
| 13854 | 0 (0, 3.33) | 0 (0, 5.21) | NA | 0.79 (0.02, 4.34) | NA |
| 13855 | 0.92 (0.02, 5.01) | 2.9 (0.35, 10.08) | NA | 0 (0, 2.89) | NA |
| 13856 | 1.83 (0.22, 6.47) | 0 (0, 5.21) | NA | 0 (0, 2.89) | NA |
| 13857 | 0.92 (0.02, 5.01) | 1.45 (0.04, 7.81) | NA | 0.79 (0.02, 4.34) | NA |
| 13858 | 0 (0, 3.33) | 0 (0, 5.21) | NA | 3.17 (0.87, 7.93) | NA |
| 13859 | 0 (0, 3.33) | 1.45 (0.04, 7.81) | NA | 0 (0, 2.89) | NA |

NA =<5 isolates, not tested

Supplementary Table 3. Proportion of isolates belonging to a particular serotype at each CSS.

| Serotype | CSS-1 | CSS-2 | CSS-1 vs CSS-2 | CSS-3 | CSS-1 vs CSS-3 |
| --- | --- | --- | --- | --- | --- |
|  | Proportion (N=109) | Proportion (N=69) | p value | Proportion (N=126) | p value |
|  | % (95% CI) | % (95% CI) |  | % (95% CI) |  |
| 3 | 4.59 (1.51, 10.38) | 0 (0, 5.21) | 0.644 | 3.17 (0.87, 7.93) | 1.000 |
| 4 | 3.67 (1.01, 9.13) | 2.9 (0.35, 10.08) | 1.000 | 0 (0, 2.89) | NA |
| 5 | 0.92 (0.02, 5.01) | 2.9 (0.35, 10.08) | NA | 0 (0, 2.89) | NA |
| 6A | 5.5 (2.05, 11.6) | 11.59 (5.14, 21.57) | 0.644 | 4.76 (1.77, 10.08) | 1.000 |
| 6B | 0.92 (0.02, 5.01) | 2.9 (0.35, 10.08) | NA | 0 (0, 2.89) | NA |
| 7F | 0.92 (0.02, 5.01) | 1.45 (0.04, 7.81) | NA | 0.79 (0.02, 4.34) | NA |
| 8 | 0 (0, 3.33) | 0 (0, 5.21) | NA | 1.59 (0.19, 5.62) | NA |
| 9L | 0 (0, 3.33) | 0 (0, 5.21) | NA | 0.79 (0.02, 4.34) | NA |
| 9V | 3.67 (1.01, 9.13) | 0 (0, 5.21) | NA | 2.38 (0.49, 6.8) | 1.000 |
| 10A | 5.5 (2.05, 11.6) | 2.9 (0.35, 10.08) | 0.974 | 4.76 (1.77, 10.08) | 1.000 |
| 10B | 2.75 (0.57, 7.83) | 1.45 (0.04, 7.81) | NA | 0.79 (0.02, 4.34) | NA |
| 11A | 2.75 (0.57, 7.83) | 2.9 (0.35, 10.08) | 1.000 | 0 (0, 2.89) | NA |
| 12F | 0 (0, 3.33) | 1.45 (0.04, 7.81) | NA | 0 (0, 2.89) | NA |
| 13 | 0.92 (0.02, 5.01) | 5.8 (1.6, 14.18) | 0.5997 | 0.79 (0.02, 4.34) | NA |
| 14 | 3.67 (1.01, 9.13) | 1.45 (0.04, 7.81) | 1.000 | 3.97 (1.3, 9.02) | 1.000 |
| 15B/15C | 12.84 (7.2, 20.61) | 11.59 (5.14, 21.57) | 1.000 | 12.7 (7.44, 19.8) | 1.000 |
| 16F | 9.17 (4.49, 16.23) | 13.04 (6.14, 23.32) | 0.974 | 12.7 (7.44, 19.8) | 1.000 |
| 17F | 2.75 (0.57, 7.83) | 0 (0, 5.21) | NA | 0.79 (0.02, 4.34) | NA |
| 18C | 0.92 (0.02, 5.01) | 0 (0, 5.21) | NA | 0 (0, 2.89) | NA |
| 19A | 5.5 (2.05, 11.6) | 7.25 (2.39, 16.11) | 1.000 | 4.76 (1.77, 10.08) | 1.000 |
| 19B | 2.75 (0.57, 7.83) | 4.35 (0.91, 12.18) | 1.000 | 3.17 (0.87, 7.93) | 1.000 |
| 19F | 5.5 (2.05, 11.6) | 5.8 (1.6, 14.18) | 1.000 | 3.17 (0.87, 7.93) | 1.000 |
| 20 | 1.83 (0.22, 6.47) | 2.9 (0.35, 10.08) | NA | 2.38 (0.49, 6.8) | 1.000 |
| 21 | 1.83 (0.22, 6.47) | 4.35 (0.91, 12.18) | 0.974 | 1.59 (0.19, 5.62) | NA |
| 23A | 2.75 (0.57, 7.83) | 2.9 (0.35, 10.08) | 1.000 | 0.79 (0.02, 4.34) | NA |
| 23B | 0.92 (0.02, 5.01) | 0 (0, 5.21) | NA | 0.79 (0.02, 4.34) | NA |
| 23F | 1.83 (0.22, 6.47) | 4.35 (0.91, 12.18) | 0.974 | 3.17 (0.87, 7.93) | 1.000 |
| 24 | 0 (0, 3.33) | 0 (0, 5.21) | NA | 2.38 (0.49, 6.8) | NA |
| 28F | 0 (0, 3.33) | 0 (0, 5.21) | NA | 0.79 (0.02, 4.34) | NA |
| 33D | 0.92 (0.02, 5.01) | 0 (0, 5.21) | NA | 3.17 (0.87, 7.93) | 1.000 |
| 34 | 11.01 (5.82, 18.44) | 1.45 (0.04, 7.81) | 0.280 | 3.97 (1.3, 9.02) | 0.239 |
| 37 | 1.83 (0.22, 6.47) | 2.9 (0.35, 10.08) | NA | 7.94 (3.87, 14.11) | 0.239 |
| 35B | 0.92 (0.02, 5.01) | 1.45 (0.04, 7.81) | NA | 2.38 (0.49, 6.8) | NA |
| 35F | 0 (0, 3.33) | 0 (0, 5.21) | NA | 2.38 (0.49, 6.8) | NA |
| 38 | 0.92 (0.02, 5.01) | 0 (0, 5.21) | NA | 0 (0, 2.89) | NA |
| 45 | 0 (0, 3.33) | 0 (0, 5.21) | NA | 7.14 (3.32, 13.13) | 0.064 |

NA =<5 isolates, not tested
